# COVID-19 Lockdown-Related Changes in Mood, Health and Academic Functioning

**DOI:** 10.3390/ejihpe11040103

**Published:** 2021-11-18

**Authors:** Pauline A. Hendriksen, Johan Garssen, Elisabeth Y. Bijlsma, Ferdi Engels, Gillian Bruce, Joris C. Verster

**Affiliations:** 1 Division of Pharmacology, Utrecht Institute for Pharmaceutical Sciences, Utrecht University, 3584 CG Utrecht, The Netherlands; p.a.hendriksen@students.uu.nl (P.A.H.); j.garssen@uu.nl (J.G.); e.y.bijlsma@uu.nl (E.Y.B.); g.m.h.engels@uu.nl (F.E.); 2 Global Centre of Excellence Immunology, Nutricia Danone Research, 3584 CT Utrecht, The Netherlands; 3 Division of Psychology and Social Work, School of Education and Social Sciences, University of the West of Scotland, Paisley PA1 2BE, UK; gillian.bruce@uws.ac.uk; 4 Centre for Human Psychopharmacology, Swinburne University, Melbourne, VIC 3122, Australia

**Keywords:** COVID-19, lockdown, online education, remote teaching, academic performance, social interactions, mood, sleep, quality of life

## Abstract

The COVID-19 pandemic lockdowns were accompanied by an abrupt transition from face-to-face education to online education. The aim of this study was to evaluate the impact of the COVID-19 pandemic on academic functioning and mood in Dutch pharmacy students and PhD candidates. A total of *n* = 341 participants completed an online survey including questions on mood and academic functioning, assessed retrospectively for before and during the COVID-19 pandemic. Overall, during COVID-19 lockdown, significantly more time was spent on academic activities, and study grades/output significantly improved. However, the overall effects were of small magnitude, and there was great variability among students, reporting either improved, unchanged or poorer academic functioning. Compared to before COVID-19, the lockdown periods were associated with significantly increased levels of stress, anxiety, depression, fatigue, and loneliness, and a significant reduction in optimism and happiness. Significant negative correlations were found between ‘performance quality’ and stress, ‘performance quality’ and fatigue, ‘study grades/output’ and stress, and between ‘study grades/output’ and fatigue. Correlations of mood and items related to academic interactions were not statistically significant. Differential effects were seen when the data was analyzed according to sex, living situation, and ethnicity, revealing that women, students living alone, and those with a migration background reported that COVID-19 lockdowns had greater negative mood effects and a more negative impact on academic functioning. Poorer sleep quality and reduced quality of life were significantly associated with reduced mood, as well as reduced academic performance quality and role satisfaction. Regression analysis revealed that being young and not having a non-Western migration background were predictors of improved performance quality. However, only being young was a significant predictor of improved study grades/output during the COVID-19 pandemic. Increased levels of stress and fatigue were significant predictors of both reduced performance quality and poorer study grades/output during the COVID-19 pandemic. In conclusion, for the sample as a whole, the transition to online education during the COVID-19 lockdown was judged as having significant positive effects on academic performance. The lockdown periods were associated with significantly reduced mood and reduced social interactions. It should be taken into account that about one third of students reported academic functioning to be poorer during the COVID-19 pandemic. This represents a substantial group of students who require more attention and guidance to make a successful transition to online education and cope with lockdown-associated stress and fatigue.

## 1. Introduction

On 11 March 2020, the World Health Organization (WHO) declared the 2019 coronavirus (COVID-19) a pandemic [1]. In order to reduce the spread of the SARS-CoV-2 virus, lockdowns were implemented, including closure of restaurants and businesses, travel restrictions, curfews, advice to quarantine when necessary, as well as the closure of schools and universities [2]. At the top of the first wave of the COVID-19 pandemic (assessed 30 March 2020 by UNESCO), 167 countries implemented school closures [3]. For higher education specifically, in order to minimize in-person contacts, part of teaching and research activities were canceled (e.g., clinical courses and internships), and many universities switched to online education platforms in order to resume educational activities [2,4].

The lockdowns and stay-at-home orders were successful in reducing the spread of the SARS-CoV-2 virus. However, students’ health and well-being were not addressed in most G20 countries [4]. Currently, a growing body of scientific literature points at associated negative psychological and health effects among university students [5,6,7]. Emerging adulthood is a transitional period in which major changes in education, living situation, and relationships are experienced. Biological, social, and developmental changes characterize this period in life, and in case these changes are not implemented successfully, this may result in the development of mental disorders such as anxiety and depression [8,9,10]. Major life events, such as a lockdown, can have a significant impact on students’ development and mental wellbeing. Several studies in student samples reported that lockdowns were associated with increased mental health complaints such as stress, anxiety, depression, and loneliness [5,7,11,12,13,14,15]. Sometimes lockdown-related mental health complaints were directly related to online education (e.g., spending too much time in front of the computer screen); in other cases, these were more clearly related to the lockdown itself (e.g., feeling lonely or depressed) [13]. A study among Malaysian students found that online education and associated concerns on academic performance as well as uncertainty about their career prospects were important factors contributing to stress during COVID-19 lockdown [14]. This was confirmed in a US study [16] that further found that other stress-inducing factors included health concerns (both for themselves and their loved ones), concentration problems, disrupted sleep, and decreased social interactions. On the other hand, a study from Kazakhstan found that mental health of medical students improved after the transition to online education [11]. In this prospective study, the prevalence of the burnout syndrome, depression, anxiety, and somatic symptoms decreased after transitioning to online teaching.

The switch to online education was a challenging change that had to be accomplished in a very short time period [17]. It has been suggested that the transition to online education has had a negative impact on academic functioning, as well as on acquiring competences and skills [2,18]. The most important challenges in the transition to online education are (1) expectations, readiness and engagement of learners; (2) ability and interest of instruction to adapt to changed requirements of online education; and (3) adjustment of course content to the online learning environment in such a way that content, pedagogy and technology support the learning activities and outcomes [19]. In addition, while technology may have offered solutions for the learning process per se, the online environment may not have adequately replaced other aspects of academic life such as interpersonal contacts. Indeed, students often reported that they miss the interactions with other students and teachers during the COVID-19 pandemic [20]. In addition, for some students, the COVID-19 measures implied an abrupt departure from their dormitories at campus, significantly impacting their social life and interactions with other students [4]. 

Some studies reported that the transition to online education had positive effects on academic functioning. A study from Saudi Arabia [21] found that 58% of basic year medical students and staff were highly satisfied with online education, and female students reported significant increases in study grades. Online education was associated with higher student achievements and resulted in improvement of the technological skills of teachers. Moreover, satisfaction with academic performance was higher in the period of online education. In Spain, the lockdown was associated with significantly improved academic performance. This effect was not only seen for courses that changed to an online format but also for teaching activities that continued in a face-to-face format [22]. These results indicated that students changed their learning strategies during the COVID-19 lockdown. In contrast to before the COVID pandemic, educational activities became a more continuous day-to-day activity during lockdown, which improved efficiency and academic performance [21].

Other studies reported a significant negative impact of COVID-19 lockdown and online education on academic functioning [5,12,13,14,15,16,23,24]. A study from Saudi Arabia also found that students and staff perceived conventional teaching as more effective and accessible than online teaching, and reported less technical difficulties, and less fraud and cheating on examinations compared to online assessments [16]. Other examples of negative aspects of online education included less-effective interactions with teachers and other students, the cancelation of practical courses, and increased possibility of cheating on exams [13]. Furthermore, studies reported online education to be associated with being less motivated [4,13]. An experimental study comparing face-to-face and online problem-based learning sessions suggests that poorer online academic performance during lockdown is related to decreased participation, communication, preparation, critical thinking and group skills [24]. Moreover, a pre-COVID-19 study reported that students prefer live online lectures above recorded lectures [25], pointing at the importance of real-time interactions between teachers and students. Bolatov et al. [11] also found that in students who reported a negative impact on academic performance, the transition to online education was associated with experiencing increased symptoms of depression and anxiety. In another study, Indonesian nursing students reported that they perceived online education as burdensome, and 46.4% of students experienced severe burnout during distance learning. They reported high levels of exhaustion, which were associated with poorer academic achievement [15]. 

Taken together, mixed results have been reported regarding the impact of the transition to online education during the COVID-19 pandemic on student well-being, academic functioning and the interaction between them. While some studies report this change was successful and online education should be continued after the COVID-19 pandemic will be ended, a greater number of studies suggest that lockdown and transition to online education was associated with poorer academic functioning, a significant reduction in social interactions with teachers and students and increased mental health problems. However, the currently available data also suggests that not every student has been equally affected by the lockdown experience [26,27]. For some students, the COVID-19 pandemic negatively impacted well-being whereas for other students being at home with family improved their well-being and academic performance [26]. Other studies reported no overall lockdown effect [27]. Results from an Indian study illustrate these differential outcomes: whereas 43% of students considered the COVID-19 lockdown as a convenient break, 60% of students stated they could not focus on their academic activities [28]. In addition, research has shown that other factors such as sex [10,29,30] and living situation [11,14] play a role in how students experienced the change to online education and coped with the lockdown situation. For example, Prowse et al. [10] reported that the negative effects on mood and academic functioning associated with the lockdown and online education were significantly more pronounced in female students compared to male students. Particularly students that lived alone during lockdown showed to be more susceptible to depression [11] and anxiety [14].

### 1.1. The COVID-19 Pandemic in the Netherlands

**Figure 1:**
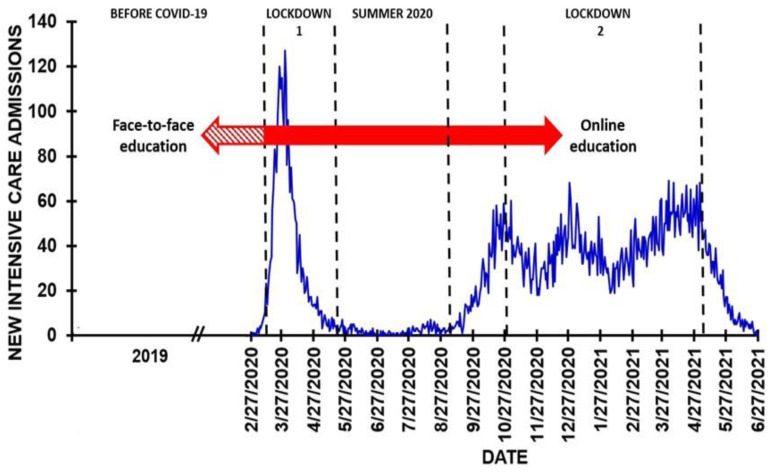
Number of COVID-19 intensive care patients in the Netherlands and related lockdown periods. Data from reference [31].

The first confirmed COVID-19 case in the Netherlands was reported on 27 February 2020. Measures to reduce the spread of SARS-CoV-2 infection by the Dutch government were taken in response to the limited Dutch intensive care capacity. Figure 1 shows that during the first half of March 2020 the demand on intensive care capacity due to COVID-19 rose exponentially. A first lockdown was enforced from the 15th of March to the 11th of May, 2020. People were advised to work from home when possible and universities switched from face-to-face to online education. On the 11th of May 2020, the lockdown ended and universities partially reopened. However, social distancing measures were still enforced, and as a result, most teaching activities remained online. Throughout the summer of 2020 there were no major COVID-19 outbreaks in The Netherlands. Academic activities were minimal during the summer and most students enjoyed the holiday season. After summer (September 2020), limited on campus education was re-instated, where universities aimed at welcoming students on campus for practical course work, research internships and once a week for regular course work. From September onward, the number of positive COVID-19 cases rose again, and a second lockdown was installed in November (2020). This lockdown lasted until April 2021. During the second lockdown, regular classes switched to online education again, whereas practical course work and research internships continued on campus.

### 1.2. Education at the Department of Pharmaceutical Sciences 

Participants of the study were students, PhD candidates and postdocs from the department of Pharmaceutical Sciences of Utrecht University, The Netherlands. Pharmacy students first follow a three-year Bachelor program, most often followed by a Master program comprising three more years of education, resulting in a PharmD degree. Within the Bachelor of Pharmaceutical Sciences, the department hosts a research-oriented international program, the College of Pharmaceutical Sciences (CPS). In addition, the Utrecht Institute of Pharmaceutical Sciences (UIPS), the research institute of the department, offers a PhD program in Drug Innovation and is responsible for the further development of postdoctoral researchers. In the academic year 2020–2021, 662 students followed the Bachelor of Pharmaceutical Sciences and 142 students the College of Pharmaceutical Sciences. A total of 458 students were registered for the Master, and 190 PhD candidates and 30 postdocs were affiliated with UIPS. Teaching at the department is mainly centered around small-scale, student-centered and context-rich teaching formats with a strong focus on collaborative learning, such as problem-based learning (e.g., case-based learning) and project- and inquiry-based learning. These types of classes are supplemented with lectures and practical course work. Before the start of the pandemic, most teaching was done on campus, although some courses had already implemented some form of computer-supported learning for, e.g., self-study and peer-feedback assignments. 

### 1.3. Aims of the Study

This study aimed to evaluate the impact of the transition to online education during COVID-19 lockdown on academic functioning and mood in Bachelor and Master students and PhD candidates from the department of Pharmaceutical Sciences at Utrecht University, The Netherlands. The lack of preparation time for this new mode of delivery as well as the inexperience with online education of both teachers and students may have impacted both the quality of teaching and academic performance of students. In addition, stay-at-home orders during the lockdown may have reduced interpersonal communication and increased negative mood including depression, loneliness, and stress. It is hypothesized that students will have experienced a decrease in overall mood during lockdown and that academic functioning will have been compromised. 

Sex differences were explored because some research has suggested that male, but not female, students perform better on campus than online [32], whereas in other studies, women reported greater difficulty adapting to online educational methods than men and also reported greater negative mood changes during lockdown periods than men [10,29,30]. Because the lockdowns may have been particularly challenging for international students who were often devoid of adequate in-person social support from family and friends [33], the data were also evaluated according to living situation (alone or together with others) and ethnicity.

## 2. Materials and Methods

This study comprised a quantitative, retrospective survey. In the first week of June 2021, an online survey was conducted among pharmacy students, PhD students, and postdocs of Utrecht University, The Netherlands. The survey was developed in SurveyMonkey, and participants were invited via email to complete the survey. The survey could be completed in Dutch or in the English language. As an incentive to complete the survey, participants could enter a drawing to win a prize of one of two 100-euro vouchers. The study was approved by the Science-Geosciences Ethics Review Board of Utrecht University (approval code: S-21525, approval date: 19 May 2021), and electronic informed consent was obtained from each participant before the start of the survey. A thorough description of the study methodology and the forthcoming dataset have been published elsewhere [34].

### 2.1. Survey Content

In addition to collecting data on demographics, retrospective assessments of mood and health correlates were made for (1) the year 2019 (the period before COVID-19), (2) the first lockdown period, (3) summer 2020 (no lockdown), and (4) the second lockdown (November 2020 to April 2021) (See Figure 1). Changes in academic functioning were assessed for the COVID-19 pandemic as a whole, in comparison with the situation before the start of the COVID-19 pandemic. 

#### 2.1.1. Demographics

Age was assessed and participants indicated whether they were student, PhD candidate or postdoc, and whether they lived alone or together with family or others (e.g., students or friends) during the COVID-19 pandemic. Ethnicity was recorded according to the definitions set by Statistics Netherlands [35]. Participants could choose between “Dutch”, “Western migration background”, or “non-Western migration background”. According to the Statistics Netherlands definition, a “Western migration background” refers to people descending from European counties (excluding Turkey), North America, Oceania, Indonesia, or Japan. A “non-Western migration background” refers to people from Africa, Latin America, and Asia (excluding Indonesia and Japan, and including Turkey).

#### 2.1.2. Mood

Mood was assessed via 1-item scales including “stress”, “anxiety”, “depression”, “fatigue”, “loneliness”, “optimism”, and “happiness”. All items were scored on a scale ranging from 0 (absent) to 10 (extreme). The 1-item scales have been validated previously [36] and applied successfully in comparable surveys [37,38,39]. The scales were completed for (1) the year 2019 (the period before COVID-19), (2) the first lockdown period, (3) summer 2020 (no lockdown), and (4) the second lockdown (November 2020 to March 2021).

#### 2.1.3. Academic Functioning

A questionnaire to assess academic functioning was developed in-house, and it focused on academic performance, social interactions, and satisfaction with academic life. Participants rated their academic functioning during the COVID-19 pandemic compared to before the start of the COVID-19 pandemic, on scales ranging from −5 (extremely worse) to +5 (extremely improved), around a midpoint of 0 (unchanged). Six items relating to academic performance were assessed. These included ‘quality’ (“overall performance quality”), ‘time’ (“amount of time invested in study/PhD candidate/postdoc project”), ‘study grades/output’ (“study grades/output”), ‘knowledge’ (“academic achievement/amount of knowledge gained”), ‘reading’ (“reading articles/text books”), and ‘writing’ (“writing assignments/articles”). Two items relating to academic interactions including “contact with teachers or supervisors” and “interactions with other students/PhD candidates/postdocs” were also evaluated. Finally, two other items relating to satisfaction with academic life were assessed and included ‘balance study-private life’ (“balance between work/study and private life”) and ‘role-satisfaction’ (“the extent you enjoy being a student/PhD candidate/postdoc”).

#### 2.1.4. Sleep and Being Active

Using a 1-item scale, ranging from 0 (absent) to 10 (extreme), “being active” was assessed. In a similar way, “sleep quality” was assessed on a scale ranging from 0 (very poor) to 10 (excellent). The 1-item scales have been validated previously [36] and applied successfully in comparable surveys [37,40,41]. The scales were completed for (1) the year 2019 (the period before the lockdown), (2) the first lockdown period, (3) summer 2020 (no lockdown), and (4) the second lockdown (November 2020 to April 2021). 

#### 2.1.5. Quality of Life

Quality of life was assessed on a scale ranging from 0 (very poor) to 10 (excellent). The scale was completed for (1) the year 2019 (the period before COVID-19), (2) the first lockdown period, (3) summer 2020 (no lockdown), and (4) the second lockdown (November 2020 to March 2021). The 1-item quality of life scale has been validated and successfully applied in previous research [36,37].

#### 2.1.6. Perceived Immune Fitness

To assess general immune status during the COVID-19 pandemic, the Immune Status Questionnaire (ISQ) was completed [42]. The ISQ consists of seven items, including “common cold”, “diarrhea”, “sudden high fever”, “headache”, “muscle and joint pain”, “skin problems (e.g., acne and eczema)”, and “coughing”. All items were scored on a 5-point Likert scale stating how often participants experienced these complaints over the past 12 months, with “never”, “sometimes”, “regularly”, “often”, and “(almost) always” as answering possibilities. The overall ISQ score ranges from 0 (poor) to 10 (excellent), with higher scores indicating a better perceived immune fitness. Previous research with the ISQ reported a Cronbach’s alpha of 0.632 and a test-retest reliability of 0.80 [42].

To assess perceived immune fitness at specific time points, a 1-item scale ranging from 0 (poor) to 10 (excellent) was used, with higher scores indicating a better perceived immune fitness [42,43,44]. The test-retest reliability of the 1-item perceived immune fitness score was 0.887 [42]. Perceived immune fitness was rated for (1) the year 2019 (the period before the lockdown), (2) the first lockdown period, (3) summer 2020 (no lockdown), and (4) the second lockdown (November 2020 to April 2021).

### 2.2. Statistical Analysis

Data were analyzed with SPSS (IBM Corp. Released 2013. IBM SPSS Statistics for Windows, Version 27.0. Armonk, NY, USA: IBM Corp.). Mean and standard deviation (SD) were computed for all variables and distributions were checked for normality with the Kolmogorov-Smirnov test and by visual inspection. Mood and academic functioning data were not normally distributed, and therefore, nonparametric tests were conducted for the statistical analysis. 

Within-subject comparisons were conducted in order to compare assessments made for four timepoints (2019, first lockdown, summer, second lockdown). This was done with the Related-Samples Friedman’s Two-Way Analysis of Variance by Ranks test. A Bonferroni’s correct was applied, and differences were considered significant if the adjusted *p* < 0.05. For the within-subjects comparison between ‘before COVID-19’ versus ‘during COVID-19’, the Related-Samples Wilcoxon Signed Ranks test was applied. Between-group comparisons for 2 groups (e.g., sex) were conducted with the Independent Samples Mann-Whitney U test. A Bonferroni’s correct was applied, and differences between the groups were considered significant if *p* < 0.007 for mood assessments and if *p* < 0.006 for academic functioning. Between-group comparisons in case there were 3 groups (e.g., education level) were made with the Kruskal-Wallis test. A Bonferroni’s correction was applied, and differences were considered significant if the adjusted *p* < 0.05. To further evaluate relationships between mood and academic performance, Spearman’s correlations were computed between the Δ scores (during COVID-19–before COVID-19). Correlations were considered significant if *p* < 0.0008, applying a Bonferroni’s correction for multiple comparisons. Separate analyses were conducted for variables that may influence study outcomes, including sex, ethnicity, living situation, and education level. Finally, binary logistic regression analyses were conducted to identify variables which significantly predict improvement or impairment of overall academic performance quality, and variables which significantly predict improvement or reduced study grades/output during the COVID-19 lockdowns. Variables included in the analyses were all demographics and all health and mood assessments. 

## 3. Results

Of the *n* = 1482 people that were invited to complete the survey, *n* = 360 participants viewed the informed consent page survey (response rate 24.3%). Of these, *n* = 358 participants provided informed consent. One participant was excluded because her age was out of range (60 years old) and 16 others did not start the survey. Demographics of the remaining *n* = 341 participants are summarized in Table 1. It is evident from Table 1 that significantly more females participated in the study than males. 

The results of mood assessments are summarized in Table 2. Compared to before COVID-19, the first lockdown was associated with significantly increased stress (*p* < 0.001), anxiety (*p* < 0.001), depression (*p* < 0.001), and loneliness (*p* < 0.001), and a significant reduction in optimism (*p* < 0.001) and happiness (*p* < 0.001). No change was found for fatigue. For the summer period, most mood ratings returned to pre-COVID-19 levels, and no significant differences were found except for reductions in levels of stress and fatigue, which were significantly lower than pre-COVID-19 rating (*p* < 0.001), and loneliness ratings that remained significantly higher compared to pre-COVID-19 (*p* = 0.020). Nevertheless, the summer period was accompanied with significantly increased ratings of optimism (*p* < 0.001). Ratings for the second lockdown were usually of greater magnitude than those for the first lockdown, suggesting that negative mood changes became more pronounced as the pandemic progressed. Compared to before COVID-19, all assessments were significantly worse for the second lockdown (*p* < 0.001). The direct comparison of the first and second lockdown revealed significantly increased ratings for stress (*p* = 0.005), depression (*p* = 0.003), fatigue (*p* < 0.001), lonely (*p* = 0.020), and decreased ratings of optimism (*p* = 0.001) and happiness (*p* = 0.040) for the second lockdown, suggesting an overall worsening of mood while the COVID-19 pandemic progressed. 

**Figure 2:**
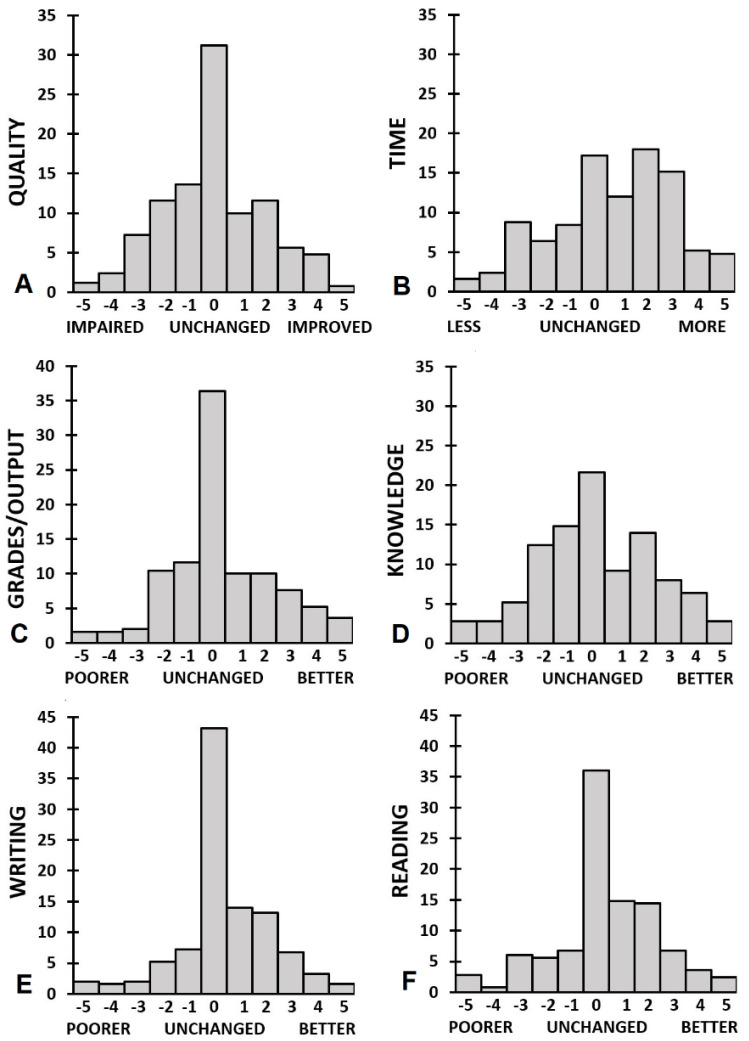
Academic performance. Distribution of ratings for (**A**) overall academic performance quality, (**B**) time, (**C**) grades/output, (**D**) knowledge, (**E**) writing, and (**F**) reading.

Figure 2 summarizes the reported impact of the COVID-19 pandemic on academic functioning. In Table 3, the data are also shown separate for Bachelor and Master students and PhD students. Scores range from −5 to +5, with negative scores implying a greater negative impact on academic functioning, and positive scores suggesting that academic performance improved during the COVID-19 pandemic. The group of postdocs was very small (*n* = 6) and therefore not considered in the analysis.

The data summarized in Table 3 reveals that for the sample as a whole, academic performance outcomes such as time invested, grades/output, reading and writing were improved significantly (*p* < 0.005) during the COVID-19 pandemic. However, Figure 2 shows a clear division among the participants: about one third report negative effects of the COVID-19 pandemic, whereas another third of the sample reports that academic performance improved during the pandemic. No significant differences were found according to education level, except for PhD students reporting a reduction in academic performance quality (*p* = 0.006) and a reduction in academic output (*p* = 0.001), compared to Bachelor students who reported improved grades and academic performance quality during the COVID-19 lockdown.

**Figure 3:**
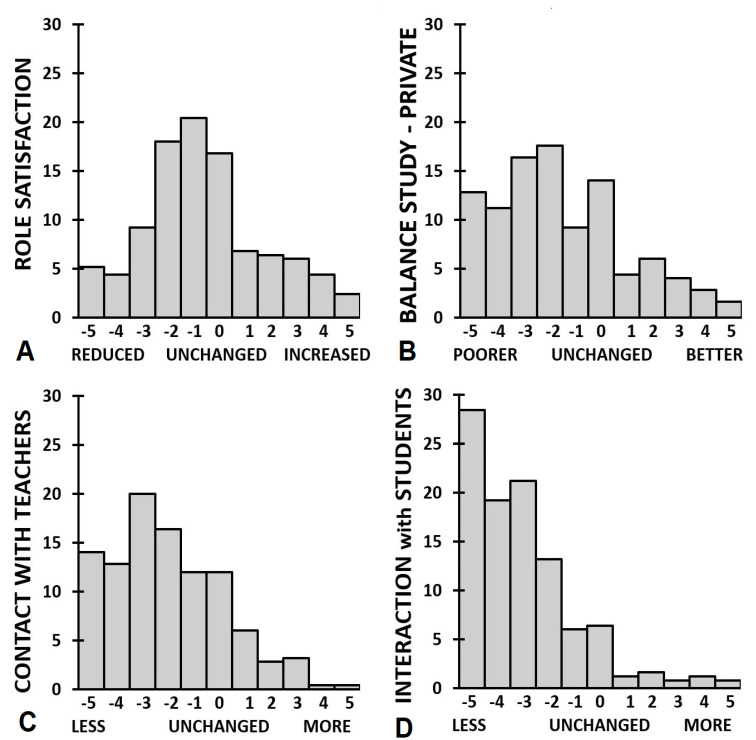
Academic interactions. Distribution of ratings for (**A**) role satisfaction, (**B**) balance study-private life (**C**) contact with teachers, and (**D**) interactions with students.

Across all education levels, significant reductions were reported for interactions with teachers and other students (See Table 3 and Figure 3C,D). Moreover, a significant reduction in role satisfaction was reported, as well as a significant disbalance between study and private life. Comparisons between education levels revealed that PhD students reported a significantly smaller reduction in contacts with teachers/supervisors than Bachelor and Master students (*p* = 0.001 and *p* = 0.034, respectively). No significant differences between education levels were seen for the reduction in interactions with other students, or the significantly distorted balance between study and private life.

Table 4 summarizes correlations between the difference scores on mood and academic functioning outcomes. Difference scores were computed as ‘‘the average of the two lockdown periods during COVID-19” minus “before COVID-19”. The rating for ‘summer 2020’ was omitted in the calculation because there was no lockdown during this period and academic activities were minimal. 

Significant negative correlations were found between ‘general performance quality’ and stress (*p* < 0.001) and fatigue (*p* < 0.001). Moreover, significant negative correlations were found between ‘study grades/output’ and stress (*p* < 0.001), and between ‘grades/output’ and fatigue (*p* < 0.001). While the correlations for these performance-related outcome variables were significant, the correlations of mood with items related to academic interactions were not statistically significant. Finally, it should be noted that while the correlation between ‘general performance quality’ and ‘study grades/output’ was highly significant (*r* = 0.709, *p* < 0.001), their correlations with ‘interactions with other students’ or ‘contact with teachers’ were not significant. Significant correlations of ‘general performance quality’ and ‘study grades/output’ with ‘balance study-private life’ (*r* = 0.234, *p* < 0.001 and *r* = 0.160, *p* = 0.011, respectively) and ‘the extent you enjoy being a student/PhD candidate/postdoc’ (*r* = 0.510, *p* < 0.001 and *r* = 0.375, *p* < 0.001, respectively) were found. 

### 3.1. Sex Differences

Mood and academic performance data according to sex are summarized in Table 5 and Table 6, respectively. 

Table 5 shows that for both men and women, the first lockdown was associated with negative mood changes. These were reduced during the lockdown-free summer period, and of greatest magnitude during the second lockdown. Although women in general reported mood changes of greater magnitude than men, after applying a Bonferroni’s correction for multiple comparisons, most sex differences were not statistically significant. 

Compared to men, women reported significantly higher levels of anxiety during the first lockdown (*p* = 0.005); however, during the second lockdown, this sex difference did not reach statistical significance (*p* = 0.009). Moreover, women reported significantly higher levels of fatigue during the first lockdown (*p* = 0.004), and although the fatigue levels were higher during the second lockdown for both men and women, this sex difference did not reach statistical significance during the second lockdown (*p* = 0.012). 

Men reported a significant reduction in contact with teachers or supervisors (*p* < 0.0001) and interactions with other (PhD) students (*p* < 0.001). The distortion in balance between work/study and private life (*p* = 0.009) did not reach statistical significance after Bonferroni’s correction was applied. In women, the effects were of the same magnitude as observed in men, but due to the larger sample size in women, more items reached statistical significance. For academic functioning, none of the direct comparisons between men and women reached statistical significance (See Table 6).

### 3.2. Ethnicity

Mood and academic performance data according to ethnicity are summarized in Table 7 and Table 8, respectively. Several ethnic differences were observed. 

Anxiety ratings of participants with a Western migration background were significantly higher than those of Dutch students for 2019 (*p* = 0.001), the first lockdown (*p* = 0.001), summer 2020 (*p* = 0.001), and the second lockdown (*p* < 0.001). For the second lockdown, anxiety ratings of participants with a Western migration background were also significantly higher than ratings of participants with a non-Western migration background (*p* = 0.024). Compared to Dutch participants, depression scores of non-Western participants were significantly higher for 2019 (*p* = 0.037), the first lockdown (*p* = 0.004), and the second lockdown (*p* = 0.012). Moreover, fatigue of non-Western participants was significantly higher than ratings by Dutch participants for both the first (*p* = 0.041) and second lockdown period (*p* = 0.041). Compared to Dutch participants, loneliness ratings for 2019 were significantly higher for participants of both Western (*p* = 0.030) and non-Western migration background (*p* = 0.031). However, paired comparisons revealed no significant differences for ethnicity during the COVID-19 pandemic. During the first lockdown, Dutch participants were significantly more optimistic than participants with a Western (*p* = 0.034) or non-Western migration background (*p* = 0.043). In 2019, Dutch participants also reported being significantly happier than participants with a Western (*p* = 0.030) or non-Western migration background (*p* = 0.005). For the first lockdown, Dutch participants remained significantly happier than participants with a Western (*p* < 0.001) or non-Western migration background (*p* = 0.002). During the second lockdown, only the difference between Dutch and non-Western participants remained significant (*p* = 0.023). 

Although these are clear mood differences between the ethnic groups, these were not translated in differential ethnic effects on academic functioning; for none of the items statistically significant differences were found between the groups (see Table 8).

### 3.3. Living Situation

Mood ratings according to living situation are summarized in Table 9. No significant differences between the groups were found. 

With regard to academic functioning (see Table 10), participants that live alone reported a reduction in academic performance quality, whereas those living with family reported an improvement. This difference was significant (*p* = 0.032). No other differences in academic functioning according to living situation were found.

### 3.4. Health Correlates

Table 11 summarizes health correlates assessed for before and during the COVID-19 pandemic. Compared to before COVID-19, the COVID-19 pandemic had a significant negative impact on perceived immune fitness (*p* < 0.001), sleep quality (*p* = 0.001), being active (*p* < 0.001), and quality of life (*p* < 0.001).

Perceived immune fitness was significantly reduced during the second lockdown compared to before COVID-19 (*p* = 0.046), the first lockdown (*p* < 0.001) and summer 2020 (*p* < 0.001). Other paired comparisons were not statistically significant for perceived immune fitness. In line, sleep quality was significantly reduced during the second lockdown compared to before COVID-19 (*p* < 0.001), the first lockdown (*p* = 0.001) and summer 2020 (*p* < 0.0001). Compared to before COVID-19, quality of life was significantly reduced during the two lockdown periods (*p* < 0.001), but not during summer 2020. Finally, compared to before COVID-19, participants reported being significantly less active during the pandemic.

The relationship of mood and health correlates is summarized in Table 12. Increased ratings of depression, fatigue and loneliness were significantly associated with reduced perceived immune fitness during the COVID-19 pandemic (*p* < 0.005).

**Figure 4:**
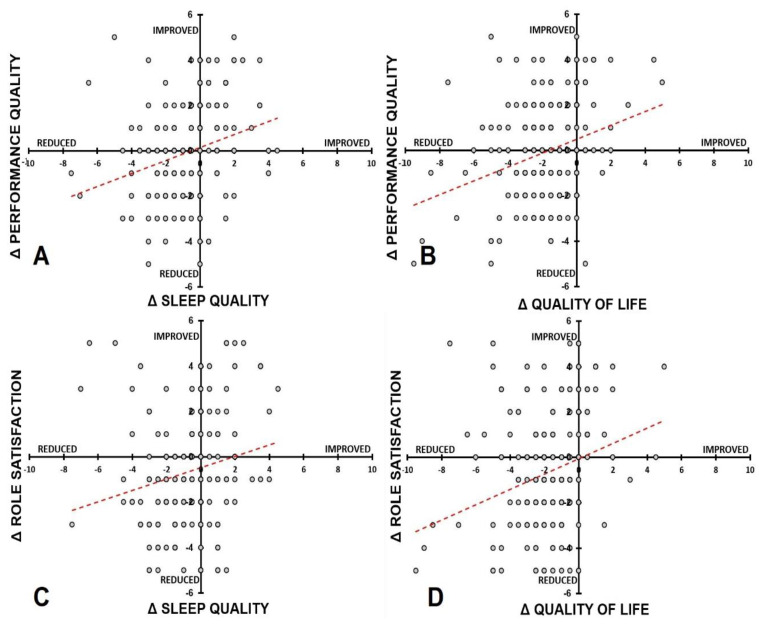
Relationship between sleep quality and quality of life with academic performance quality and role satisfaction. Spearman’s correlations for difference scores are shown between (**A**) sleep quality and performance quality, (**B**) quality of life and performance quality, (**C**) sleep quality and role satisfaction, and (**D**) quality of life and role satisfaction.

Correlations of immune outcomes and academic functioning outcomes did not significantly correlate with each other (see Table 13). Moreover, being active did not show strong correlations with mood and academic functioning. In contrast, both sleep quality and quality of life were significantly correlated with all mood assessments except optimism and being happy, and they also showed significant correlations with academic performance quality (*p* < 0.001) and role satisfaction (*p* < 0.001) (See Figure 4).

### 3.5. Predicting Improved Versus Poorer Academic Functioning

Binary regression analyses were conducted to determine which variables predict improved or reduced academic performance quality, and increased or lower study grades/output. The results are summarized in Table 14.

The analyses revealed that stress and fatigue were significant predictors of reduced academic performance quality and lower study grades/output. Improved academic performance quality was significantly predicted by being young and not having a non-Western migration background. Increased study grades/output was significantly predicted only by being younger.

## 4. Discussion

The COVID-19 pandemic was accompanied by an abrupt transition from face-to-face education to online education. The aim of this study was to evaluate the impact of the COVID-19 pandemic on academic functioning and mood in Dutch pharmacy students, PhD candidates, and postdocs. Overall, during the COVID-19 lockdown, significantly more time was spent on academic activities and study grades/output significantly improved. However, the overall effects were of small magnitude, and there was great variability among students, reporting either improved, unchanged or poorer academic functioning. Compared to before COVID-19, the lockdown periods were associated with significantly increased levels of stress, anxiety, depression, fatigue, and loneliness, and a significant reduction in optimism and happiness. Regression analysis revealed that being young and not having a non-Western migration background were predictors of improved performance quality, but that only being young was a significant predictor of improved study grades/output during the COVID-19 pandemic. Increased levels of stress and fatigue were significant predictors of both reduced performance quality and poorer study grades/output during the COVID-19 pandemic.

It has previously also been suggested that distance learning could replace traditional face-to-face courses [45,46], and these positive effects on academic performance have also been reported for the COVID-19 pandemic [47]. However, when interpreting the data, it should be taken into account that about one third of students reported that academic functioning was poorer than before COVID-19. This represents a substantial group of students that requires more attention and guidance to make a successful transition to online education, and cope with lockdown-associated mood and health complaints.

It appeared that being young was the best predictor of academic performance improvement, both in self-reported overall quality and study grades/outcomes. There may be several explanations for this observation. It might be that younger individuals are more flexible in changing learning and educational strategies. It is important to note that especially first-year students not only needed to cope with the abrupt transition to online education, but also with the transition from high school to university. In addition, particularly the older Master students and PhD candidates may have specifically suffered from restrictions on practical courses (e.g., workplace learning), laboratory closures and research restrictions. As such, the COVID-19 pandemic may have had a greater negative impact on academic performance of older students, PhD candidates, and postdocs. A smaller impact on academic performance quality improvement was found for not having a non-Western migration background. This may be related to the specific subjective perception of performance quality by individuals with a non-Western migration background because ethnicity was not a significant predictor of the more objective indicator ‘study grades/output’. Academic performance impairment, both quality and study grades/output, were predicted by having increased levels of stress and fatigue. Previous research has already shown that levels of stress correlate with study output [11,13,15]. The current study adds to the field by showing that high levels of stress and fatigue can actually negatively affect academic performance. 

The mixed observation of both impaired and improved academic functioning is in line with pre-COVID-19 assessments. These studies also reported mixed outcomes when comparing online to face-to-face teaching, in which online teaching either resulted in poorer academic performance [48], equivalent performance [49,50], or better academic functioning [26]. The difference in outcomes during the COVID-19 pandemic may also be related to the fact that most universities initially focused on the conversion of teaching content to online platforms. This process had to be accomplished in a very short time period. These time constraints implied that universities did not necessarily adapt their conventional face-to-face pedagogy into an adequate online pedagogy [5]. Further, students were also not always prepared for an abrupt change to online education. Although the availability of online education platforms brings several advantages (e.g., flexibility and mobility), having to learn in an online environment may also require significant unforeseen adjustments from students [51]. First of all, other research within Utrecht University indicated that from an international perspective, major differences between countries and between students exist regarding to what extent students possess digital skills and have access to digital technologies with adequate connection to the internet [52]. Because The Netherlands is amongst the top five of European countries regarding internet use by people 16–74 years old (94% in 2020) [52], it is likely that the students within the study population were quite well equipped to adjust to the online learning environment. It can be expected that the negative impact of this transition to online education is even stronger in countries where students have less well-developed digital skills.

It must be noted that living situation, education level, and sex were not among the significant predictors in the regression analysis. Indeed, no significant differences were found for these variables on academic functioning. Moreover, except for increased levels of fatigue among female students during the first lockdown period, no significant differences were found for the predictors of academic performance impairment (i.e., stress and fatigue) according to sex or living situation. Research on these variables in relation to academic performance during the COVID-19 pandemic is limited and yielded mixed results. For example, whereas a study from Taiwan found that on campus courses were more suitable for male students, no significant difference between conventional and online teaching methods were found among female students [31], whereas another study from Kazakhstan [11] reported no sex differences in academic functioning after the transition to online education. Studies do show that mood and health effects among female students are usually more pronounced compared to those in men. This was also the case in the current study, but due to the stringent Bonferroni’s correction, except for anxiety during the first lockdown, the sex differences did not reach statistical significance.

### 4.1. Limitations 

The current study has several limitations that are important to take into account when interpreting the data. Firstly, a convenience sample of Dutch students, PhD candidates and postdocs completed the survey. It is unknown to what extent these individuals are representative for students from other disciplines or students from different countries. To further investigate this, a comparative study in Argentina is currently in progress. Secondly, the assessments were made by retrospective self-report. Therefore, recall bias may have influenced the study outcomes. For future studies, it is advisable to use a prospective study design, making real-time assessments. Thirdly, while the 1-item mood and perceived immune fitness assessments have been validated previously [36,42], the scale assessing academic functioning was developed specifically for this study. Of importance while developing the scale was that it would be brief and clear; it is important that future research confirms the validity and reliability of the scale by directly comparing it with such traditional questionnaires and actual study grades. Finally, we did ask participants whether they had been tested and had COVID-19. This is important to know because it can have a direct impact on mood, perceived immune fitness, and academic performance. However, we did not ask participants when exactly they tested positive. Therefore, it was decided not to incorporate this data in the statistical analysis because it remains unknown whether the infection took place during the lockdowns when academic activities were conducted or during the summer holiday season. It is recommended that future research records the data of testing and disease progress over time in order to enable relating this data to other study outcomes.

### 4.2. Implications

This study adds to the literature in that it showed that among the various mood changes and COVID-19 pandemic correlates, specifically increased levels of stress and fatigue are significant contributors to reduced academic performance quality and poorer study grades/output. Stress and fatigue should therefore be monitored in students, and preventive measures could be enforced to reduced stress and fatigue. For example, the current study found a significant correlation between sleep quality and academic functioning. Informing students that adequate sleep is important to reduce daytime fatigue (and stress), including providing guidance on sleep hygiene, can therefore be an important prevention strategy. Moreover, providing guidance on alternative ways to relax, despite being in lockdown at home, may be helpful to reduce stress. For example, being outdoors and active have positive effects on mood. If the stress is specifically caused by home confinement that prevent adequate academic functioning (e.g., a small room size, living together with others), Utrecht university and other universities offer study spaces at the university. However, it is essential that universities can identify students at risk that are in need of these facilities. Students may not be aware of this possibility, nor will all of them proactively contact university to seek help. Therefore, monitoring stress and fatigue of students by university (e.g., by mentors of student counselors) is of great importance, both during the COVID-19 pandemic and thereafter.

### 4.3. Future Research

It is important to note that there are different educational methodologies and pedagogic strategies that may have different advantages and disadvantages when implementing online education. The effectiveness of these methodologies in the transition to online education during the COVID-19 pandemic and engaging students in learning should be evaluated and compared by future research. In terms of lessons learned, the current data support the notion that transactional distance and affective proximity matter to make pedagogy successful [53]. As such, the significant reduction of social interactions during the COVID-19 pandemic has been reported to dehumanize educational processes. In this context, it is important to critically reconsider the future of education [53,54]. Should we simply return (unchanged) to traditional face-to-face education, continue online education, or a mix of both? As both modes of education have advantages and disadvantages, considering a most perfect mix between the two seems ideal. In dept evaluation and comparison of the two modes of education—including the specifically used education methodologies, as well as the choice of supportive technology—is therefore warranted. 

## 5. Conclusions

The aim of this study was to evaluate the impact of the COVID-19 pandemic on academic functioning and mood in Dutch pharmacy students, PhD candidates, and postdocs. For the sample as a whole, the transition to online education during COVID-19 lockdown was judged as having significant positive effects on academic performance. However, the lockdown periods were also associated with significantly reduced mood and reduced social interactions. Regression analysis revealed that being young and not having a non-Western migration background were predictors of improved performance quality but that only being young was significant predictor of improved study grades/output during the COVID-19 pandemic. Increased levels of stress and fatigue were significant predictors of both reduced performance quality and poorer study grades/output during the COVID-19 pandemic.

## Figures and Tables

**Table 1:** Demographics.

Variable	Study Outcome
*n*	341
Male/Female	85 (24.9%)/256 (75.1%)
Age (years)	23.0 (4.2)
*Ethnicity*	
—Dutch	223 (65.8%)
—Western migration background	37 (10.9%)
—Non-Western migration background	79 (23.3%)
*Living situation*	
Alone	41 (12.0%)
Together with others (students, friends)	131 (38.4%)
Together with family	169 (49.6%)
*Educational level*	
—Bachelor pharmacy or CPS	179 (52.5%)
—Master pharmacy	117 (34.3%)
—PhD candidate	39 (11.4%)
—Postdoctoral researcher	6 (1.8%)

Results for age, weight, height, and BMI are presented as mean (SD); other variables as number (%). Abbreviation: BMI = body mass index, SD = standard deviation, CPS = college of pharmaceutical sciences (a research Bachelor).

**Table 2:** Mood.

Variable	Before COVID-19	First Lockdown	Summer 2020 (No Lockdown)	Second Lockdown
Stress	5.0 (2.2)	5.6 (2.6) ^a^	3.7 (2.5) ^a,b^	6.1 (2.5) ^a,b,c^
Anxiety	2.5 (2.6)	3.8 (3.0) ^a^	2.7 (2.6) ^a,b^	3.7 (3.2) ^a,c^
Depression	2.1 (2.5)	2.9 (2.9) ^a^	2.0 (2.5) ^b^	3.6 (3.1) ^a,b,c^
Fatigue	4.2 (2.6)	4.3 (2.7)	3.1 (2.6) ^a,b^	5.5 (2.7) ^a,b,c^
Lonely	1.7 (2.1)	3.7 (2.9) ^a^	2.3 (2.4) ^a,b^	4.4 (3.2) ^a,b,c^
Optimistic	7.1 (1.9)	5.9 (2.1) ^a^	6.7 (1.8) ^b^	5.4 (2.1) ^a,b,c^
Happy	7.1 (1.8)	5.8 (2.0)	7.1 (1.8)	5.4 (2.0)

Mean (SD) are shown. Significant differences (adjusted *p*-values < 0.05, applying a Bonferroni’s correction for multiple comparisons) are indicated as follows: ^a^ = significantly different from ‘before COVID-19’, ^b^ = significant difference from the ‘first lockdown’, ^c^ = significant difference from ‘summer 2020’.

**Table 3:** Academic functioning during the COVID-19 pandemic.

Academic Functioning	Overall	Bachelor Students	Master Students	PhD Students
Quality	0.0 (2.0)	0.3 (2.0)	−0.2 (2.0)	−0.9 (1.8) ^a^
Time	0.7 (2.4) *	0.9 (2.5) *	0.6 (2.2)	0.5 (2.5)
Grades/Output	0.4 (2.0) *	0.8 (2.0) *	0.1 (2.0)	−0.6 (1.9) ^a^
Knowledge	0.2 (2.3)	0.3 (2.5)	0.0 (2.2)	0.2 (1.7)
Reading	0.4 (2.0) *	0.3 (2.1)	0.3 (2.2)	0.7 (1.2)
Writing	0.4 (1.8) *	0.6 (1.8) *	0.2 (1.8)	0.2 (1.6)
Contact with teachers	−2.1 (2.2) *	−2.3 (2.3) *	−2.0 (2.0) *	−1.0 (1.6) *^,a,b^
Interactions with students	−3.0 (2.1) *	−2.9 (2.2) *	−3.3 (1.9) *	−2.6 (1.6) *
Balance study-private life	−1.5 (2.5) *	−1.5 (2.6) *	−1.4 (2.5) *	−2.0 (2.4) *
Role satisfaction	−0.6 (2.4) *	−0.5 (2.5)	−0.6 (2.2)	−1.0 (2.2)

Mean (SD) are shown. Significant changes relative to ‘before COVID-19’ (Wilcoxon Signed Ranks test (*p* < 0.005, after Bonferroni’s correction) are indicated by *. Significant differences between the groups (*p* < 0.002, after Bonferroni’s correction) are indicated as follows: ^a^ = significantly different from Bachelor students, ^b^ = significantly different from Master students.

**Table 4:** Correlations between mood changes and academic functioning during the COVID-19 pandemic.

Variable	Stress	Anxiety	Depression	Fatigue	Lonely	Optimistic	Happy
Quality	−0.261 *	−0.198	−0.159	−0.321 *	−0.114	0.188	0.210
Time	−0.122	0.066	−0.023	−0.167	0.015	0.084	0.053
Grades/Output	−0.234 *	−0.096	−0.070	−0.252 *	−0.003	0.051	0.076
Knowledge	−0.079	−0.035	−0.004	−0.197	−0.099	0.137	0.083
Reading	−0.051	−0.111	−0.025	−0.043	−0.063	0.094	0.089
Writing	−0.019	0.040	−0.035	−0.078	−0.116	0.131	0.105
Contact with teachers	−0.107	−0.112	−0.043	−0.079	−0.131	0.163	0.114
Interactions with students	−0.089	−0.118	−0.001	0.088	−0.107	0.068	0.117
Balance study-private life	−0.259 *	−0.358 *	−0.214	−0.155	−0.245 *	0.222 *	0.257 *
Role satisfaction	−0.314 *	−0.291 *	−0.203	−0.277 *	−0.241	0.273 *	0.272 *

Spearman’s correlations between difference scores (average score of the two COVID-19 lockdowns—before COVID-19) are shown. Significant correlations (*p* < 0.001, after Bonferroni’s correction) are indicated by *.

**Table 5:** Mood before and during COVID-19 according to sex.

Mood	Before COVID-19		First Lockdown		Summer 2020		Second Lockdown	
Variable	Men	Women	Men	Women	Men	Women	Men	Women
Stress	4.7 (2.3)	5.2 (2.1)	5.0 (2.6)	5.8 (2.6) ^a^	3.5 (2.2) ^b^	3.8 (2.6) ^a,b^	5.5 (2.6) ^a,c^	6.3 (2.4) ^a,b,c^
Anxiety	2.1 (2.6)	2.6 (2.6)	2.9 (2.8) ^a^	4.1 (3.0) *^,a^	2.2 (2.3)	2.9 (2.6) ^b^	2.8 (3.0)	4.0 (3.2) ^a,c^
Depression	2.1 (2.7)	2.1 (2.5)	2.8 (2.9)	2.9 (2.9) ^a^	2.0 (2.5)	1.9 (2.5) ^b^	3.6 (3.1) ^a,c^	3.6 (3.1) ^a,b,c^
Fatigue	3.5 (2.7)	4.4 (2.5)	3.4 (2.5)	4.6 (2.7) *	2.7 (2.2)	3.3 (2.7) ^a,b^	4.7 (3.0) ^a,b,c^	5.8 (2.5) ^a,b,c^
Lonely	1.6 (2.0)	1.8 (2.1)	3.4 (2.9) ^a^	3.8 (2.9) ^a^	2.1 (2.4) ^b^	2.3 (2.5) ^b^	4.3 (3.2) ^a,c^	4.4 (3.2) ^a,c^
Optimistic	6.9 (2.2)	7.1 (1.8)	6.0 (2.2) ^a^	5.8 (2.1) ^a^	6.5 (2.1)	6.8 (1.7) ^b^	5.4 (2.4) ^a,c^	5.4 (2.0) ^a,b,c^
Happy	6.6 (2.1)	7.2 (1.7)	5.7 (2.0) ^a^	5.8 (2.0) ^a^	6.6 (2.2) ^a^	7.3 (1.6) ^b^	5.1 (2.2) ^a,c^	5.5 (1.9) ^a,c^

Mean and standard deviation (SD) are shown. Significant sex differences (*p* < 0.007, after Bonferroni’s correction for multiple comparisons) are indicated by *. Other significant differences (adjusted *p*-values < 0.05, applying a Bonferroni’s correction for multiple comparisons) are indicated as follows: ^a^ = significantly different from ‘before COVID-19’, ^b^ = significant difference from the ‘first lockdown’, ^c^ = significant difference from ‘summer 2020’.

**Table 6:** Academic functioning during the COVID-19 pandemic according to sex.

Academic Functioning	Men	Women
Quality	−0.1 (1.9)	0.0 (2.0)
Time	0.1 (2.5)	1.0 (2.3) *
Grades/Output	0.3 (1.9)	0.4 (2.1)
Knowledge	0.2 (2.3)	0.2 (2.3)
Reading	0.3 (2.0)	0.4 (2.0)
Writing	0.2 (2.0)	0.5 (1.7) *
Contact with teachers	−2.0 (2.2) *	−2.0 (2.2) *
Interactions with students	−2.9 (2.2) *	−3.0 (2.0) *
Balance study-private life	−1.0 (2.8)	−1.7 (2.4) *
Role satisfaction	−0.6 (2.3)	−0.6 (2.4) *

Mean (SD) are shown. Significant changes relative to ‘before COVID-19’ (Wilcoxon Signed Ranks test) (*p* < 0.005, after Bonferroni’s correction for multiple comparisons) are indicated by *. No significant differences between men and women were found.

**Table 7:** Mood before and during COVID-19 according to ethnicity.

Variable	Ethnicity	Before COVID-19	First Lockdown	Summer 2020	Second Lockdown
**Stress**	Dutch	4.9 (2.1)	5.3 (2.5)	3.5 (2.3) ^a,b^	6.0 (2.4) ^a,b,c^
	Western	5.6 (2.0)	6.4 (2.5)	4.3 (2.9) ^b^	6.9 (2.4) ^a,c^
	Non-Western	5.1 (2.5)	5.9 (3.0) ^a^	4.0 (2.7) ^b^	6.0 (2.5) ^a,c^
**Anxiety**	Dutch	2.2 (2.5)	3.3 (2.8) ^a^	2.3 (2.3) ^b^	3.1 (2.9) ^a,c^
	Western	3.9 (2.6) *	5.4 (2.9) *^,a^	4.2 (2.5) *^,b^	6.0 (3.3) *^,a,c^
	Non-Western	2.6 (2.7)	4.3 (3.3) ^a^	3.2 (2.9) ^b^	4.0 (3.2) ^†,a^
**Depression**	Dutch	1.8 (2.5)	2.4 (2.8) ^a^	1.8 (2.4) ^b^	3.1 (3.0) ^a,b,c^
	Western	2.5 (2.4)	3.4 (2.4)	2.0 (2.3)	4.6 (3.3) ^a,c^
	Non-Western	2.7 (2.7) *	3.8 (3.2) *^,a^	2.5 (2.8) ^b^	4.5 (3.1) *^,a,c^
**Fatigue**	Dutch	3.9 (2.5)	4.0 (2.5)	2.9 (2.5) ^a,b^	5.2 (2.6) ^a,b,c^
	Western	4.6 (2.6)	4.7 (2.8)	3.2 (2.6)	6.3 (2.6) ^c^
	Non-Western	4.7 (2.7)	5.1 (3.1) *	3.9 (2.8) ^b^	6.2 (2.7) *^,a,c^
**Lonely**	Dutch	1.4 (1.8)	3.5 (2.8) ^a^	2.2 (2.3) ^a,b^	4.1 (3.1) ^a,c^
	Western	2.4 (2.2) *	4.3 (3.1) ^a^	2.5 (2.8)	5.5 (3.3) ^a,c^
	Non-Western	2.3 (2.5) *	4.0 (3.2) ^a^	2.3 (2.8) ^b^	4.7 (3.2) ^a,c^
**Optimistic**	Dutch	7.4 (1.7)	6.2 (2.0) ^a^	6.9 (1.8) ^b^	5.5 (1.9) ^a,b,c^
	Western	6.5 (2.2)	5.3 (2.0) *^,a^	6.5 (1.9)	4.9 (2.6) ^a,c^
	Non-Western	6.5 (2.2) *	5.3 (2.4) *^,a^	6.4 (1.8)	5.3 (2.2) ^a,c^
**Happy**	Dutch	7.4 (1.6)	6.2 (1.8) ^a^	7.3 (1.6) ^b^	5.7 (1.8) ^a,b,c^
	Western	6.4 (2.1) *	4.9 (1.7) *^,a^	6.5 (2.0) ^b^	4.6 (2.6) ^a,c^
	Non-Western	6.5 (2.1) *	5.1 (2.2) *^,a^	6.8 (2.1) ^b^	5.0 (2.0) *^,a,c^

Mean (SD) are shown. Significant differences (adjusted *p* values < 0.05, after Bonferroni’s correction) are indicated as follows: * = significantly different from Dutch participants, ^†^ = significant difference between Western and non-Western migration background. Other differences (adjusted *p*-values < 0.05, applying a Bonferroni’s correction) are indicated as follows: ^a^ = significantly different from ‘before COVID-19’, ^b^ = significant difference from the ‘first lockdown’, ^c^ = significant difference from ‘summer 2020’.

**Table 8:** Academic functioning during the COVID-19 pandemic according to ethnicity.

Academic Functioning	Dutch	Western Migration Background	Non-Western Migration Background
Quality	0.0 (1.9)	−0.7 (2.0)	0.2 (2.3)
Time	0.7 (2.3) *	1.0 (2.2)	0.7 (2.8)
Grades/Output	0.4 (1.8)	0.1 (2.0)	0.6 (2.6)
Knowledge	0.1 (2.1)	0.5 (2.0)	0.3 (2.9)
Reading	0.4 (1.8)	1.2 (2.2)	0.0 (2.4)
Writing	0.3 (1.7)	1.0 (1.6)	0.3 (2.2)
Contact with teachers	−2.2 (2.0) *	−1.0 (2.0)	−1.9 (2.6) *
Interactions with students	−3.2 (1.9) *	−2.3 (2.3) *	−2.8 (2.4) *
Balance study-private life	−1.6 (2.4) *	−1.6 (3.1)	−1.4 (2.6) *
Role satisfaction	−0.6 (2.2) *	−1.0 (2.5)	−0.4 (2.6)

Mean (SD) are shown. Significant changes relative to ‘before COVID-19’ (*p* < 0.005, after Bonferroni’s correction for multiple comparisons) are indicated by *. No significant differences were found between the groups.

**Table 9:** Mood before and during COVID-19 according to living situation.

Variable	Living Situation	Before COVID-19	First Lockdown	Summer 2020	Second Lockdown
**Stress**	Alone	5.1 (2.5)	5.1 (3.0)	3.5 (2.8)	6.3 (2.8)
	With others	4.8 (2.1)	5.2 (2.6)	3.5 (2.3)	5.9 (2.6)
	With family	5.3 (2.2)	6.0 (2.5)	3.9 (2.6)	6.2 (2.3)
**Anxiety**	Alone	3.4 (3.0)	4.1 (3.2)	3.3 (3.0)	4.2 (3.8)
	With others	2.3 (2.5)	3.4 (2.9)	2.4 (2.5)	3.4 (3.0)
	With family	2.4 (2.5)	4.0 (3.0)	2.8 (2.5)	3.8 (3.2)
**Depression**	Alone	3.0 (2.7)	3.4 (3.0)	2.7 (2.8)	4.5 (3.4)
	With others	1.7 (2.4) *	2.3 (2.6)	1.6 (2.4)	3.4 (3.0)
	With family	2.2 (2.6)	3.2 (3.1)	2.1 (2.5)	3.6 (3.1)
**Fatigue**	Alone	4.5 (2.4)	4.2 (2.8)	2.9 (2.4)	5.5 (2.8)
	With others	3.9 (2.6)	3.9 (2.5)	3.0 (2.5)	5.6 (2.7)
	With family	4.4 (2.6)	4.7 (2.8)	3.3 (2.7)	5.6 (2.7)
**Lonely**	Alone	2.5 (2.5)	3.8 (3.1)	2.5 (2.9)	5.0 (3.5)
	With others	1.3 (1.7)	3.4 (2.7)	1.8 (2.1)	4.0 (3.0)
	With family	1.8 (2.2)	3.9 (3.0)	2.6 (2.5)	4.6 (3.2)
**Optimistic**	Alone	7.2 (1.8)	6.3 (2.3)	7.1 (1.6)	5.6 (2.3)
	With others	7.4 (1.9)	6.1 (2.1)	7.0 (1.7)	5.5 (1.9)
	With family	6.8 (2.0)	5.6 (2.1)	6.5 (2.0)	5.2 (2.2)
**Happy**	Alone	7.0 (1.6)	5.9 (2.0)	7.2 (2.0)	5.8 (2.1)
	With others	7.4 (1.8)	6.1 (1.8)	7.5 (1.5)	5.5 (1.9)
	With family	6.8 (1.9) ^†^	5.5 (2.1)	6.8 (1.9) ^†^	5.2 (2.0)

Mean (SD) are shown. Significant differences for living situation (adjusted *p* values < 0.05, after Bonferroni’s correction for multiple comparisons) are indicated as follows: * = significantly different from living alone, ^†^ = significant difference between living with others and living with family.

**Table 10:** Academic functioning during the COVID-19 pandemic according to living situation.

Academic Functioning	Alone (*n* = 30)	With Others (*n* = 99)	With Family (*n* = 121)
Quality	−0.9 (2.2)	−0.2 (1.7)	0.4 (2.1) ^†^
Time	0.0 (2.5)	0.8 (2.2) *	0.8 (2.5) *
Grades/Output	−0.3 (2.3)	0.2 (1.6)	0.7 (2.2) *
Knowledge	−0.2 (2.5)	0.1 (1.9)	0.4 (2.6)
Reading	0.3 (1.9)	0.5 (1.7) *	0.2 (2.3)
Writing	0.4 (2.0)	0.5 (1.4) *	0.3 (2.0)
Contact with teachers	−1.4 (2.5)	−2.0 (2.0) *	−2.1 (2.2) *
Interactions with students	−3.3 (1.8) *	−3.0 (1.9) *	−2.9 (2.2) *
Balance study-private life	−0.6 (2.9)	−1.8 (2.2) *	−1.6 (2.7) *
Role satisfaction	−0.6 (2.7)	−0.8 (1.8) *	−0.4 (2.7)

Mean (SD) are shown. Significant changes relative to ‘before COVID-19’ (*p* < 0.005, after Bonferroni’s correction for multiple comparisons) are indicated by *. Significant differences between living alone and living with family are indicated by ^†^.

**Table 11:** Health correlates.

Variable	Before COVID-19	First Lockdown	Summer 2020 (No Lockdown)	Second Lockdown
Perceived immune fitness	7.5 (1.7)	7.2 (1.9)	7.4 (1.7)	6.9 (1.9) ^a,b,c^
Sleep quality	7.0 (1.8)	6.8 (2.0)	7.2 (1.7) ^b^	6.2 (2.1) ^a,b,c^
Being active	6.2 (2.6)	4.7 (2.9) ^a^	5.4 (2.7) ^a,b^	4.5 (2.8) ^a,c^
Quality of life	7.7 (1.4)	6.2 (1.9) ^a^	7.4 (1.5) ^b^	5.9 (2.0) ^a,c^

Mean (SD) are shown. Significant differences (adjusted *p*-values < 0.05, applying a Bonferroni’s correction for multiple comparisons) are indicated as follows: ^a^ = significantly different from ‘before COVID-19’, ^b^ = significant difference from the ‘first lockdown’, ^c^ = significant difference from ‘summer 2020’.

**Table 12:** Relationship between health correlates, mood and academic functioning.

Variable	ISQ	Perceived Immune Fitness	Sleep Quality	Being Active	Quality of Life
Stress	0.025	−0.119	−0.302 *	−0.035	−0.295 *
Anxiety	−0.104	−0.038	−0.261 *	−0.026	−0.276 *
Depression	−0.024	−0.225 *	−0.224 *	−0.169	−0.305 *
Fatigue	−0.033	−0.241 *	−0.406 *	−0.022	−0.267 *
Lonely	−0.139	−0.213 *	−0.197 *	−0.194 *	−0.291 *
Optimistic	0.047	0.132	0.187	0.076	0.459 *
Happy	0.010	0.164	0.268 *	0.134	0.134

Spearman’s correlations between difference scores (average score of the two COVID-19 lockdowns—before COVID-19) are shown. Significant correlations (*p* < 0.005, after Bonferroni’s correction) are indicated by *.

**Table 13:** Relationship between health correlates and academic functioning.

Variable	ISQ	Perceived Immune Fitness	Sleep Quality	Being Active	Quality of Life
Quality	−0.111	0.073	0.295 *	−0.052	0.219 *
Time	−0.088	0.007	0.108	−0.060	0.107
Grades/Output	−0.154	0.060	0.127	−0.068	0.108
Knowledge	0.040	0.070	0.123	0.020	0.153
Reading	0.089	−0.050	−0.091	−0.039	0.055
Writing	−0.020	−0.039	−0.036	−0.196 *	0.076
Contact with teachers	0.139	0.036	0.043	0.057	0.129
Interactions with students	0.123	−0.047	−0.027	−0.002	0.161
Balance study-private life	0.061	−0.038	0.154	−0.044	0.221 *
Role satisfaction	0.052	0.067	0.231 *	−0.049	0.282 *

Spearman’s correlations between difference scores (average score of the two COVID-19 lockdowns—before COVID-19) are shown. Significant correlations (*p* < 0.005, after Bonferroni’s correction) are indicated by *.

**Table 14:** Variables predicting academic performance quality and study grades/output.

		95% Confidence Interval for Odds Ratio		
Model	B	Lower	Odds Ratio	Upper
**Academic Performance Quality**				
*Improvement*				
Constant	2.42			
Age	−0.12 *	0.81	0.89	0.97
Non-Western ethnicity	−1.50 **	0.10	0.32	0.99
*R*^2^ = 0.05 (Cox-Snell) 0.07 (Nagelkerke). Model X^2^ (3) = 12.7 *p* = 0.005				
*Impairment*				
Constant	−1.08			
Stress	0.26 **	1.08	1.29	1.54
Fatigue	0.26 **	1.30	1.30	1.52
*R*^2^ = 0.13 (Cox-Snell), 0.18 (Nagelkerke). Model X^2^ (2) = 34.63, *p* < 0.001				
**Study grades/output**				
*Increased*				
Constant	1.69			
Age	−0.10 *	0.84	0.91	0.98
*R*^2^ = 0.03 (Cox-Snell), 0.04 (Nagelkerke). Model X^2^ (1) = 6.92, *p* = 0.009				
*Lower*				
Constant	−1.47			
Stress	0.24 *	1.06	1.27	1.53
Fatigue	0.22 **	1.06	1.25	1.46
*R*^2^ = 0.10(Cox-Snell), 0.15 (Nagelkerke). Model X^2^ (2) = 25.88, *p* < 0.001				

Note: * *p* < 0.05, ** *p* < 0.01.

## Data Availability

The data is published open access in the journal MDPI Data and available online as supplement to reference [34].
